# Association between integrative and complementary health practices and use of dental services among older adults in Brazil: a cross-sectional study, 2019

**DOI:** 10.1590/S2237-96222022000300007

**Published:** 2022-10-17

**Authors:** Aneiza Simoní Lucas, Maria Laura Braccini Fagundes, Orlando Luiz do Amaral, Gabriele Rissotto Menegazzo, Jessye Melgarejo do Amaral Giordani

**Affiliations:** 1 Universidade Federal de Santa Maria, Departamento de Estomatologia, Santa Maria, RS, Brazil

**Keywords:** Primary Health Care, Oral Health, Complementary Therapies, Cross-Sectional Studies

## Abstract

**Objective:**

To analyze association between participation in integrative practices and regular use of dental services in Brazilian older adults.

**Methods:**

This was a cross-sectional study based on secondary data from the 2019 National Health Survey. All older adults aged 60 years and over were included. The study outcome was regular dental service use. Poisson regression models were used to estimate crude and adjusted prevalence ratios (PRs) and their respective at confidence intervals 95% (95%CI).

**Results:**

A total of 22,728 older adults were analyzed. Most were female (55.5%), reported that they were White (51.3%), had incomplete primary education (47.0%); 7.0% (95%CI 6.8;7.5) had used some form of integrative practice and 34.3% (95%CI 33.2;35.4) had used their dental service regularly. Individuals who used integrative practices had higher prevalence of dental service use even after adjusting the model (PR = 1.15; 95%CI 1.07;1.23).

**Conclusion:**

Among Brazilian older adults use of integrative practices was associated with regular use of dental services.

**Study contributions**

**Main results**

Frequency of regular use of dental services among elderly people who used integrative and complementary practices was higher than among their peers.

**Implications for services**

These practices, guaranteed by the Brazilian National Health System (SUS), can be a strategy for stimulating better health-related behaviors, such as regular visits to the dentist, as well as reinforcing the principle of comprehensive care.

**Perspectives**

Bringing together integrative practices and geriatric dentistry can benefit both service users and professionals responsible for care, providing a more affectionate, empathetic and participatory clinical relationship, as well as individual diagnosis and therapy procedures.

## Introduction

Despite the recognized importance of oral health as part of people’s overall health and well-being, a large part of Brazilians do not use dental services.^1^ Use of health services is the result of a series of complex causes, which relate to socio-demographic and economic issues as well as to morbidity profiles and the availability of health services.^2^ Predisposing factors regarding health service use, such as beliefs, personal health practices, diet, exercise and self-care, can have an effect on the perceived need for care and consequently influence the pattern of service use.^3^ According to results from the 2019 National Health Survey (*Pesquisa Nacional de Saúde* - PNS), less than 30% of elderly people had seen a dentist in the year prior to the survey interview.^1^ Oral health is essential for quality of life, whether for chewing and swallowing food, or for self-esteem and psychological well-being.^4^

Integrative and complementary health practices comprise a set of services and techniques not covered by traditional medicine.^5^ Such practices favor care focused on health and not disease, the quest for harmony between an individual and their natural and social environment, stimulating subjectivity to prevent disease and promote health, aiming at individual comprehensive care.^5^ In Primary Health Care (PHC), their relevance lies in the therapeutic pluralism necessary for the complex management of the family approach and the community approach to health, which assumes both longitudinal and comprehensive care.^6^ Such premises are especially important in care provided to the elderly population. In the face of a predominantly biomedical, curative and fragmented care model, the limitations of the effectiveness of which manifest themselves in the epidemiological pattern of diseases,^7^ we are witnessing slow but gradual growth of integrative practices as a strategy for changing health paradigms.^8^

Observing differentiated patterns of care and understanding the determinants of longevity with quality of life, can contribute positively to the aging process.^9^ Integrative practices are forms of health care that work on the different senses involving the human being, stimulating self-knowledge, instigating and recovering the notion of quality of life, as well as co-responsibility in the health-disease-care process.^10^ Therefore, it is plausible to assume that these practices have also influenced the demand for health services and oral health care.

The objective of this study was to analyze association between participation in integrative practices and regular use of dental services in Brazilian older adults.

## Methods

### Design

This was a retrospective cross-sectional study, which used secondary data from Brazil’s second National Health Survey (PNS 2019), conducted between August 2019 and March 2020 by the Brazilian Institute of Geography and Statistics (IBGE) in partnership with the Ministry of Health, covering all of Brazil’s Federative Units.

The data used in the present study were obtained from the IBGE website (https://www.ibge.gov.br/estatisticas/sociais/saude.html), accessed on May 26, 2021.

### Background

The PNS is a population-based survey and is representative of the Brazilian population that lives in permanent private residences.^11^ The first edition of the survey took place in 2013, aiming to collect information on the health determinants, conditioning factors and health needs of the Brazilian population. In 2019 Brazil had approximately 211 million inhabitants and the number of elderly people in the country had reached 32.9 million, maintaining the trend of population aging. Even so, the use of dental services in this age group remained below 30%.^1^

### Participants

Sampling was carried according to conglomerates, in three selection stages: (i) census tracts were used as primary sampling units; (ii) households were the units in the second stage; and finally, within each sampled household, (iii) the participants were selected using equiprobable sampling.^11^ The survey target population was comprised of individuals aged 15 years or older. One resident was randomly selected in each selected household, based on the list of eligible residents obtained at the time of the interview.^11^

In this study, the objective of which was to study only the elderly population, data from all research participants aged 60 years or older were analyzed, and no exclusion criteria were applied.

### Variables

- Outcome: regular use of dental services (last visit: up to 1 year ago; more than 1 year ago);
- Main exposure: use of integrative and complementary practices (yes; no).

**Figure 1:**
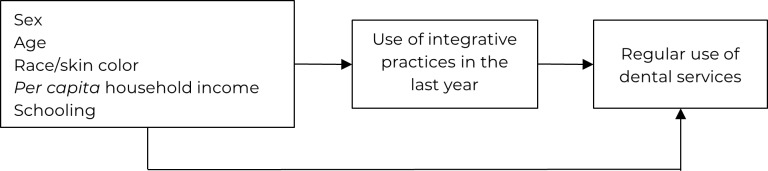
Theoretical model of the relationship between use of integrative practices and regular use of dental services, Brazil, 2019

The conceptual model (Figure 1) that guided the choice of confounding variables was specified prior to data analysis and was based on previous studies on the social determinants of health^12^ and service use.^3^ The confounding variables included:

- sex (male; female);
- age groups (collected quantitatively according to years of age and then categorized into: 60-69; 70-79; ≥ 80);
- race/skin color (Indigenous; mixed race; Asian; Black; White);
- *per capita* household income (collected quantitatively in BRL and then categorized into five income quintiles: 1^st^; 2^nd^; 3^rd^; 4^th^; 5^th^); and
- schooling (no schooling; incomplete elementary education; complete elementary education; incomplete high school education; complete high school education; incomplete higher education; complete higher education).

### Data source and measurement

IBGE organized and coordinated the fieldwork for the primary study. Data collection was conducted through interviews in the participants’ homes, where those selected answered an individual structured questionnaire. The interviews were conducted using mobile collection devices programmed with the survey questionnaire.^11^ All variables included in this study were self-reported by the participants.

### Bias control

All data collectors in the primary study were trained and calibrated to understand, in detail, the entire survey. Instructional material was prepared to guide the field team, seeking to minimize measurement bias.^11^

### Study size

In order to define the sample size with a sufficient level of precision for the parameters of interest, we used some of the indicators from the first edition of the PNS, carried out in 2013: data on chronic non-communicable diseases, violence, use of health services, having a health insurance plan, health-related behaviors, among others.^11^ The calculation considered the following criteria: estimation of frequencies with the desired level of precision, using 95% confidence intervals; effect of the sampling plan; number of selected households per primary sampling unit; and frequency of households with people in the age group of interest.^11^

### Statistical methods

Data analysis was performed using Stata software, version 14.0 (College Station, TX), considering the sample weight due to the complex sampling plan (survey module). 

Initially we described the sample characteristics, frequencies and respective 95% confidence intervals (95%CI) of the regular use of dental services according to the independent variables. Frequency of use of integrative practices according to the type of practice was also described. Associations with regular use of dental services were estimated by applying Poisson regression models, observing the crude and adjusted prevalence ratios (PRs) and their respective 95%CIs; and 5% significance levels, as per Pearson’s chi-square test. Multiple analysis was adjusted for demographic and socioeconomic factors. All variables were input to the model simultaneously.

### Ethical aspects

This study used secondary unrestricted public domain data, with no identification of participants, so that the confidentiality of the data was preserved. Although the study was not submitted to an internal institutional Research Ethics Committee, all ethical efforts were made to ensure confidentiality and compliance with National Health Council Resolution No. 466, dated December 12, 2012. Notwithstanding, the 2019 PNS, from which the data used in the research were derived, was approved by the National Research Ethics Committee/National Health Council under Opinion No. 3,529,376, issued on August 23, 2019, as per Certificate of Submission for Ethical Appraisal No. 11713319.7.0000.0008. All those who participated in the 2019 PNS signed an Informed Consent Form prior to being interviewed. The data and document files can be obtained from the IBGE website (https://www.ibge.gov.br/estatisticas/sociais/saude.html).

## Results

We analyzed data on 22,728 elderly individuals. Most respondents were female (55.5%), reported that they were White (51.3%) and had incomplete elementary education (47.0%). The highest frequency was of *per capita* family income was found in the third income quintile (29.0%). Only 7.0% (95%CI 6.8;7.5) of the elderly used integrative or complementary practices and of these, 46.8% (95%CI 43.0;50.7) used dental services on a regular basis. Overall, 34.3% (95%CI 33.2;35.4) of the elderly we assessed used dental services on a regular basis (Table 1).

**Table 1:** Description of the characteristics of the sample (n=22,728), frequencies and respective 95% confidence intervals of regular use of dental services, according to independent variables, Brazil, 2019

Variables	n (%)	Regular use of dental services
		% (95%CI^a^)
**Sex**		
Male	10,193 (44.5)	33.6 (32.0;35.2)
Female	12,535 (55.5)	34.9 (33.5;36.2)
**Age (in yers)**		
60-69	12,555 (54.8)	40.4 (39.0;41.9)
70-79	7,157 (31.1)	28.9 (27.2;30.6)
≥ 80	3,016 (14.1)	22.1 (19.9;24.5)
**Race/skin color^b^**		
Indigenous	165 (0.5)	19.9 (12.9;29.5)
Mixed race	10,001 (36.7)	26.8 (25.4;28.3)
Asian	204 (1.3)	46.2 (36.1;56.7)
Black	2,455 (10.2)	26.1 (23.5;28.9)
White	9,901 (51.3)	41.1 (39.5;42.8)
***Per capita* household income (quintiles)^b^**		
1^st^	1,325 (4.9)	24.7 (21.2;28.6)
2^nd^	3,372 (12.8)	21.0 (18.9;23.2)
3^rd^	7,041 (29.0)	21.7 (20.3;23.2)
4^th^	5,651 (27.3)	33.1 (31.3;35.1)
5^th^	5,336 (26.0)	57.7 (55.5;59.9)
**Schooling**		
No schooling	4,717 (16.6)	14.1 (12.7;15.6)
Incomplete elementary education	10,270 (47.0)	25.9 (24.6;27.3)
Complete elementary education	1,427 (6.8)	39.5 (35.3;43.8)
Incomplete high school education	584 (2.6)	35.7 (30.2;41.6)
Complete high school education	3,029 (13.9)	50.7 (47.9;53.4)
Incomplete higher education	293 (1.5)	63.1 (54.3;71.1)
Complete higher education	2,400 (11.6)	70.5 (67.6;73.3)
**Use of integrative practices in the last year**		
No	21,090 (93.0)	33.4 (32.3;34.5)
Yes	1,638 (7.0)	46.8 (43.0;50.7)

a) 95%CI: 95% confidence interval; b) Missing data due to participants not answering.

Phytotherapy was the most used type of integrative practice among those included in the survey (61.2%; 95%CI 58.1;64.2), followed by acupuncture (30.5%; 95%CI 27.3;33.9) and homeopathy (15.9%; 95%CI 13.3;18.9) (Table 2). 

**Table 2:** Integrative practice frequency and respective 95% confidence interval, per type of practice (n = 1,638), Brazil, 2019

Integrative practice	% (95%CI^a^)^b^
Phytotherapy	61.2 (58.1;64.2)
Acupuncture	30.5 (27.3;33.9)
Homeopathy	15.9 (13.3;18.9)
Meditation	8.5 (6.6;10.8)
Auriculotherapy	6.8 (4.8;9.5)
Yoga	5.7 (4.4;7.5)
Tai chi chuan	1.2 (0.6;2.4)
Integrative community therapy	0.9 (0.5;1.5)
Other	3.1 (2.1;4.6)

a) 95%CI: 95% confidence interval. Note: The participants could answer "Yes" for more than one practice they had used in the last year. That is why the sum of the values exceeds 100%.

Frequency of regular dental service use was higher in individuals who made use of integrative practices in the last year, both in the crude analysis (PR = 1.40; 95%CI 1.28;1.52) and after adjustment for demographic and socioeconomic variables (PR = 1.15; 95%CI 1.07;1.23) (Table 3).

**Table 3:** Crude and adjusted prevalence ratios and respective 95% confidence intervals of the association between regular use of dental services and use of integrative practices (n = 22,728), Brazil, 2019

Variable	Crude PR^a^ (95%CI^b^)	p-value^c^	Adjusted^d^ PR^a^ (95%CI^b^)	p-value^c^
**Use of integrative practices in the last year**				
No	1.00	< 0.001	1.00	< 0.001
Yes	1.40 (1.28;1.52)		1.15 (1.07;1.23)	

a) PR: Prevalence ratio; b) 95%CI: 95% confidence interval; c) Pearson’s chi-square test; d) Adjusted for demographic and socioeconomic factors.

## Discussion

Frequency of regular dental service use was higher among those who used integrative practices than among their peers. The findings of the present study make it possible to indicate that individuals who are more inclined to take care of their overall well-being, and for this reason use integrative practices, are also more proactive regarding their general and oral health, and thus seek dental services regularly.

This study has limitations, including its cross-sectional design, which limits causal inferences. It is possible that recall bias may have occurred since all the variables collected were self-reported. Despite their low frequency, the Asian and Indigenous race/skin color categories were kept in the analysis to avoid possible selection bias. Although the use of integrative practices is also considered to be an indicator of self-care, it should be noted that objective variables referring to self-care in health were not included due to limitations in the variables available in the database. On the other hand, standing out among this study’s potentialities is that the PNS 2019 is comprised of a nationally representative sample, which provides robustness to the results. As far as the study’s authors are aware, this is the first evaluative study of the relationship between the use of integrative practices and the use of dental services by elderly Brazilians.

Despite the fact that the Brazilian population is aging increasingly, use of dental services by the elderly continues to be low.^1^ This population requires qualified listening and generally has sequelae arising from the accumulation of oral diseases throughout their lives.^13^ It is also preferable that health workers act in a welcoming manner, taking into account psychological and social aspects of service users, seeking to understand suffering and illness from the perspective of service users.^14^ Integrative practices are therapeutic techniques that act in a complementary manner to biomedical rationality,^15^ and can be a strategy for strengthening personal autonomy.^16^ They can, therefore, serve as a stimulus for seeking dental care on a regular basis. Routine visits to the dentist enable disease prevention and minimize the complexity of procedures, unlike making emergency visits when oral health problems arise.^17^ Access to and use of dental services on a regular basis are essential for the elderly population, in order to minimize the impacts resulting from demands accumulated throughout their lives.^18^

The integrative practices most used by the elderly people interviewed were phytotherapy, acupuncture and homeopathy. Given the effects of these practices on anxiety and stress,^19^ it is possible that they also have beneficial potential for those who suffer from anxiety related to dental procedures, and may complement the care of these individuals by providing care that is more humanized and relaxed.^20^ The objective of using these therapies can be to actively include individuals in treatment decisions, improve their coping and sense of well-being.^21^ The use of integrative practices can be an important component of oral health self-care,^22^ and can motivate people to seek routine dental care, as shown by this study.

The use of these practices has been explored as a means of supporting treatment of chronic conditions,^23^ which are generally more prevalent in the elderly population. Assessment of the effects of different integrative practices on people with cancer with pain and anxiety,^23^ and stress symptoms,^24^ found psychological responses such as relaxation and decreased pain and anxiety,^23^ decreased stress levels,^24^ as well as improved emotional and spiritual well-being.^23^ Use of certain integrative practices also points to beneficial effects in reducing abusive use of antibiotics.^25^ Regarding care of the elderly, integrative practices can induce greater social interaction, boost self-esteem and stimulate the performance of daily activities,^26^ these being attitudes and behaviors that are often impaired in the lives of these subjects.^27^

Use of integrative practices appears to have positive impacts on health, considering the psychological, physical and emotional dimensions of service users.^19^ This influence may be due to the fact that these practices involve approaches that seek to stimulate disease prevention and health recovery through soft technologies, including systems and resources that emphasize friendly listening, the creation of bonds and the integration of individuals in the context in which they live.^16^ The health-disease process is therefore viewed in a comprehensive way, aimed at all-round promotion of care and, especially, encouragement of self-care. This also appears to have repercussions on oral health care, which is reflected in the regular use of this service. 

When oral health professionals apply the systemic approach proposed by integrative practices, their intention is to diagnose and treat using an approach that goes beyond physical symptoms, and also relate them to the biopsychosocial aspects of the individual’s context.^28^ Their use, alongside the practice of geriatric dentistry, regardless of the cause to be treated, can raise the health worker-service user relationship to levels of humanization that contribute to the excellence of results. It is known that in the past people who are elderly today were attended to in consulting rooms where there were no technologies capable of providing stress-free care, which often results in anxiety prior to dental treatment.^29^ In this sense, integrative practices can be potential allies in management of anxiety caused by these procedures, given their association with reduction of stress and anxiety symptoms.^24^ As they involve approaches that use soft technologies, integrative practices can avoid excessive medicalization and unnecessary interventions, helping to overcome a merely biomedical model.^30^

We found association between use of integrative practices and regular use of dental services by elderly Brazilians. These practices, already guaranteed by the Brazilian National Health System (SUS), can be a strategy for encouraging the adoption of better health-related behaviors, such as regular use of dental services.
